# Emerging Biomarkers in Bladder Cancer Identified by Network Analysis of Transcriptomic Data

**DOI:** 10.3389/fonc.2018.00450

**Published:** 2018-10-12

**Authors:** Matteo Giulietti, Giulia Occhipinti, Alessandra Righetti, Massimo Bracci, Alessandro Conti, Annamaria Ruzzo, Elisabetta Cerigioni, Tiziana Cacciamani, Giovanni Principato, Francesco Piva

**Affiliations:** ^1^Department of Specialistic Clinical and Odontostomatological Sciences, Polytechnic University of Marche, Ancona, Italy; ^2^Department of Clinical and Molecular Sciences, Polytechnic University of Marche, Ancona, Italy; ^3^Department of Urology, Bressanone/Brixen Hospital, Bressanone, Italy; ^4^Department of Biomolecular Sciences, University of Urbino “Carlo Bo”, Fano, Italy; ^5^Unit of Pediatric and Specialistic Surgery, United Hospitals, “G.Salesi”, Ancona, Italy; ^6^Department of Life and Environmental Science, Polytechnic University of Marche, Ancona, Italy

**Keywords:** WGCNA, bladder cancer, tumor biomarkers, gene expression, heterogeneity

## Abstract

Bladder cancer is a very common malignancy. Although new treatment strategies have been developed, the identification of new therapeutic targets and reliable diagnostic/prognostic biomarkers for bladder cancer remains a priority. Generally, they are found among differentially expressed genes between patients and healthy subjects or among patients with different tumor stages. However, the classical approach includes processing these data taking into consideration only the expression of each single gene regardless of the expression of other genes. These complex gene interaction networks can be revealed by a recently developed systems biology approach called Weighted Gene Co-expression Network Analysis (WGCNA). It takes into account the expression of all genes assessed in an experiment in order to reveal the clusters of co-expressed genes (modules) that, very probably, are also co-regulated. If some genes are co-expressed in controls but not in pathological samples, it can be hypothesized that a regulatory mechanism was altered and that it could be the cause or the effect of the disease. Therefore, genes within these modules could play a role in cancer and thus be considered as potential therapeutic targets or diagnostic/prognostic biomarkers. Here, we have reviewed all the studies where WGCNA has been applied to gene expression data from bladder cancer patients. We have shown the importance of this new approach in identifying candidate biomarkers and therapeutic targets. They include both genes and miRNAs and some of them have already been identified in the literature to have a role in bladder cancer initiation, progression, metastasis, and patient survival.

## Introduction

Bladder cancer (BCa) is the ninth most prevalent malignant disease globally, with more than 400,000 new cases diagnosed each year, especially in males and elderly, and the thirteenth most common cause of cancer death. Five-year survival rate for early stage BCa patients reaches 95.7%, while for metastatic patients it is just 5% (1). Recent advancements in therapy include immunotherapy with PD-1 antibody pembrolizumab and the PD-L1 antibody atezolizumab which have yielded better results compared to chemotherapy (2). The most common BCa type is transitional cell carcinoma, also called urothelial carcinoma, while squamous cell carcinoma and adenocarcinomas are rare. For the majority of new BCa patients, non-muscle-invasive BCa (NMIBC) is diagnosed. This low-grade tumor often recurs and in about 20% of cases it progresses to high grade, i.e., to the muscle-invasive BCa (MIBC) which is more likely to develop metastases (3). In addition, molecular data, including chromosomal aberrations, mutation rates, presence of mutated tumor suppressor genes, gene, and miRNA expression levels have led to the definition of specific molecular subtypes. This depicts BCa as a molecularly and clinicopathologically heterogeneous disease. Diagnosis can be based on cystoscopy, microscopy, voided urinary cytology, blood detection in the urine and assessment of urine-based tumor markers, such as complement factor H-related protein (BTA test) and nuclear matrix protein 22 (NMP22) (4). However, the sensitivity and specificity of these markers decrease in the presence of inflammatory cells and other contaminating cells in the sample (5). Therefore, the identification of new biomarkers could improve diagnostic or prognostic performance of BCa tests. Also small extracellular vesicles released by BCa are currently being investigated, since they are known to be involved in cancer growth, progression and metastatic spread and the molecules contained in these vesicles may be potential BCa biomarkers (6, 7).

An effective method to discover biomarkers is transcriptome profiling, by microarrays or more recently by RNA-seq, that allows the determination of differentially expressed genes and non-coding RNAs under different conditions. For example, the comparison of the expression levels of thousands of genes in healthy vs. cancer tissue samples, or among different tumor stages or tumors under different treatments, has led to the identification of several differentially expressed genes. They could represent candidate therapeutic targets or biomarkers for tumor onset, progression, or prognosis. However, over the past few years, a major drawback has emerged regarding differential expression analysis. In particular, differentially expressed genes are treated individually and this does not allow the identification of co-regulation mechanisms among them. Highlighting these gene interactions is important, because they are often altered in complex genetic disorders like cancer (8–10). This emerging systems biology method focuses on the analysis of gene regulation alterations allowing a better understanding of cancer onset and its progression and the identification of critical cancer driver genes. Moreover, these clusters of co-expressed genes, that constitute the regions of a complex gene network, often correspond to cellular pathways (8–10).

Currently, a widely used approach to process gene expression data and investigate network alterations is the Weighted Gene Co-expression Network Analysis (WGCNA), that draws gene networks where the connections among pairs of genes are identified and weighted based on their correlated expression levels across multiple samples (11). Briefly, after processing the expression profiles into weighted connections, WGCNA can identify the network topology and, by using the topological overlap dissimilarity as the measure of distance among genes, it allows the identification of sub-networks, called modules [for further details see (12)]. Therefore, only highly co-expressed genes (i.e., connected with strong weights in the network) can compose the gene modules. It is also possible to relate these modules to clinical traits of interest. For example, regarding the comparison of two distinct networks deriving from tumor and normal gene expression data, WGCNA can identify the modules and genes belonging to them that reflect the regulatory alterations related to transcriptional changes. In particular, the most interconnected genes in a module (hub genes) are often functionally important and thus they could play a key role in cancer and represent candidate diagnostic and prognostic biomarkers or potential therapeutic targets (13–15).

## Emerging BCa biomarkers identified by the WGCNA method

In this review, we focused on all studies where the WGCNA method was applied to analyze gene expression data deriving from BCa samples (Table 1).

**Table 1:** Hub genes and miRNAs detected by different authors using WGCNA method.

**Comparison**	**Number and source of samples**	**Expression data source**	**Genes**	**Involved pathways**	**References**
Different BCa stages	93 BCa tissue samples	NCBI GEO microarray dataset GSE31684	COL3A1, COL5A2, FBN1, POSTN	Extracellular matrix (ECM) organization, ECM-receptor interaction, regulation of actin cytoskeleton, cell adhesion, focal adhesion	(16)
Different BCa stages	165 primary BCa tissue samples	NCBI GEO microarray dataset GSE13507	AURKB, BUB1B, CCNB2, CDC45, CENPA, CEP55, KIF2C, KIF4A, KIF15, NUSAP1, PRC1, UBE2C (only diagnostic); AEBP1, CDC25B, COL5A2, MMP11, TK1, TPX2 (only prognostic); CDCA3, CDCA8, CENPF, FOXM1, TOP2A (both diagnostic and prognostic)	Cell cycle, nuclear division, chromosome segregation, organelle fission	(17)
SCCB vs. normal	75 tumor cells and 18 normal cells from the tissue of one patient	NCBI SRA single cell RNA-seq SRP078083	ARHGAP15, BCAR3, CACNA2D3, CENPH, CTNND2, DOHH, GCC2, HERC2, LINC00189, NLK, OR9Q1, PCSK6, POU2F3, SCN2A, TUBGCP2	Spliceosome complex, VEGF, MAPK, neurotrophic signaling, and cell cycle pathways	(18)
BCa vs. normal	9 BCa and 9 normal tissue samples ^*^	NCBI GEO microarray dataset GSE3167	ATF7, CER1, CYP1A2, GDF9, KCNIP1, LRRC15, PTPRJ, TRPM3	Response to stimulus, regulation of localization, gamma-hexachlorocyclohexane degradation, fatty acid metabolism, adherens junction, tryptophan metabolism, and Wnt signaling pathways	(19)
NMIBC vs. MIBC	8 NMIBC and 11 MIBC tissues^*^	NCBI GEO microarray dataset GSE37317	ATP2A2, BCAP31, BRD2, CYP3A5, DCAF8, LRRC37A2, MEIS3P1, POLR2A, PURA, SRPK2, TRAK1, UBE2I, UPF3A, VPS13D, WDFY3, ZZEF1	Regulation of mitotic cell cycle, cell cycle phase, organelle organization, negative regulation of programmed cell death, DNA replication, DNA recombination, mRNA splicing, cellular localization, B cell receptor signaling pathway, Ras pathway	(20)
BCa vs. normal	418 BCa and 19 normal tissue samples	TCGA miRNA-Seq dataset BLCA	miR-1-1, miR-1-2, miR-28, miR-133a-1, miR-133a-2, miR-133b, miR-139, miR-143, miR-145, miR-195, miR-548ba, miR-3199-2, miR-6507	Cell proliferation, regulation of cell growth, regulation of actin cytoskeleton, proteoglycans in cancer, focal adhesion, Wnt, PI3K-Akt, MAPK, and p53 signaling pathways	(21)

*BCa, bladder cancer; SCCB, squamous cell carcinoma of bladder; MIBC, muscle-invasive BCa; NMIBC, non-muscle-invasive BCa*.

* *Note that WGCNA has been performed using too few samples, indeed at least 20 samples are required for a robust WGCNA analysis and to obtain reliable results*.

Recently, WGCNA was applied on publicly available microarray gene expression data deriving from 93 BCa patients in order to identify genes related to different tumor stages (Ta, T1, T2, T3, and T4) and therefore to suggest potential prognostic biomarkers (16). A network module was highly correlated with tumor progression and further processing was performed in order to identify which cellular pathways were represented by the module genes. In general, since not all genes of a pathway are present in a module, the term “enrichment” is used to indicate how significantly a pathway overlaps with a module. A functional enrichment analysis is usually performed on a list of genes in order to reveal these enriched pathways. The identified module was significantly enriched in genes belonging to important biological processes (Table 1). Within this module, four hub genes were identified: COL3A1, COL5A2, FBN1, and POSTN and, by using independent expression datasets, the COL3A1 (Collagen type III α1 chain) gene was validated as both a diagnostic and prognostic biomarker. In fact, it was upregulated in BCa tissues compared to normal ones and its high expression strongly correlated with tumor progression, shorter overall survival (OS) and disease-free survival (DFS) times. While there are few literature data about COL5A2 and FBN1 genes, the role of POSTN (periostin) in BCa has been widely investigated. In particular, high grade BCa showed very low levels of POSTN gene and the artificial restoration of its expression suppressed cell invasiveness and metastasis (22). It was also shown that the decrease of invasiveness and the inhibition of the epithelial-to-mesenchymal transition (EMT) were due to bladder-specific upregulation of the E-cadherin expression by periostin (23).

Lately, microarray gene expression data of 165 primary BCa samples were analyzed by WGCNA in order to identify genes correlated with TNM staging and OS (17). In total, 11 modules correlated with TNM staging and they were enriched in genes belonging to cell proliferation associated pathways (Table 1). A filtering step using protein-protein interaction network information collected in STRING tool (https://string-db.org/) resulted in 17 genes with a potential role in BCa (Table 1). Moreover, 11 hub genes could be considered as prognostic biomarkers, since their lower expression was associated with better OS of BCa patients. Some identified hub genes had been previously investigated. In particular, the high expression of AURKB (Aurora Kinase B), implicated in cancer through development of aneuploidy and chromosomal instability, correlated with advanced BCa stages (24). Similarly, the mitotic checkpoint protein BUB1B, known to contribute to chromosomal instability, was over-expressed in advanced BCa stages and correlated with high cell proliferation (25). High expression levels of CDC25B (Cell division cycle 25B) were associated with advanced stages, recurrence and poor prognosis in BCa patients (26). Cyclin B2 (CCNB2) expression level was higher in cancer than in normal bladder mucosa and its downregulation inhibited cell migration, invasion, and metastatic abilities (27). Also the role of FOXM1 (Forkhead box M1) has been widely investigated, indeed it was found to be a reliable prognostic biomarker for MIBC (28) and its high expression level correlated with TNM stage, histological grade, metastases, and poor prognosis in BCa patients, whereas its down-regulation through miR-24-1 inhibited cell proliferation, migration, and invasion (29, 30). Since CCNB2 and FOXM1 suppression resulted in BCa inhibition, they can be considered as reliable therapeutic targets and deserve further exploration. Moreover, MMP11 (Matrix metalloproteinase-11) overexpression correlated with very aggressive phenotypes (advanced pT status, nodal metastasis, high histological grade) and with unfavorable clinical outcomes (31). Serum concentration of TK1 (Thymidine kinase 1) gene was associated with tumor stage, degree of invasion, and metastasis (32). TOP2A (Topoisomerase-IIA) free DNA in urine has been identified as a diagnostic biomarker and its levels can also distinguish NMIBC from MIBC (33). Its over-expression has also been associated with high-grade and high-stage BCa and with high rates of recurrence in NMIBC (34). Moreover, TOP2A protein levels have been identified as a predictor of DFS times (35). TPX2 (microtubule nucleation factor) gene was upregulated in tumor tissues compared to normal bladder samples and it was strongly associated with pT status, high histological grade, lymph node metastasis, and shorter OS time. Indeed its overexpression promoted proliferation and tumorigenicity and suppressed apoptosis (36). UBE2C (Ubiquitin conjugating enzyme E2) upregulation was associated with high BCa stages, presence of lymphovascular invasion, progression to MIBC, and it has been suggested as a biomarker of unfavorable prognosis (37, 38).

In an experiment where single-cell transcriptomics was applied to squamous cell carcinoma of urinary bladder (SCCB), 75 tumor cells and 18 normal cells were isolated from cancer and normal control fresh resected tissues from one patient (18). Then, gene expression analysis was performed by single-cell RNA-seq. WGCNA analysis identified five large modules enriched in genes belonging to several cancer-associated pathways (Table 1). While some of the identified hub genes (Table 1) have rarely been reported in previous cancer studies, some of them have already been suggested as BCa biomarkers. For example, CTNND2 (Catenin delta 2) gene is frequently amplified in BCa, with high copy numbers (39). Moreover, copy number variations of PCSK6 (Proprotein convertase subtilisin/kexin type 6) gene have been reported to be a prognostic marker for NMIBC progression (40). Intriguingly, POU2F3 (POU class 2 homeobox 3) is a transcription factor expressed in stratified squamous epithelia and related to squamous epithelial stratification (41), so it could play a role in squamous cell BCa (SCCB).

Microarray gene expression profiles of 9 normal bladder and 9 transitional cell carcinoma tissue samples have been analyzed by the WGCNA method (19). A differential co-expression network analysis was carried out and eight hub genes were identified (Table 1). Interestingly, among the identified hub genes, the rs762551 polymorphism in CYP1A2 (a cytochrome P450 family member) gene was associated with decreased BCa risk (42). Molecular mechanisms explaining this association are still undetermined, however since it lies in the first intron of the gene, it could alter pre-mRNA splicing processing at 5′UTR level, transcription regulation, or protein folding (43–51).

Recently, different gene expression profiles between NMIBC and MIBC have been investigated using public microarray expression data from 8 and 11 snap frozen cancer tissues, respectively (20). WGCNA analysis showed significant correlations between three modules and the tumor stage. In particular, these modules were enriched in genes mainly involved in cell cycle (Table 1). Among them, 16 hub genes have been identified (Table 1). Therefore, they can be involved in BCa progression from NMIBC to MIBC phenotype. Many hub genes have been previously suggested as candidate diagnostic or prognostic BCa biomarkers. For example, TRAK1 (trafficking kinesin protein 1) gene has been identified as a favorable prognostic marker, since its low level expression was associated with poorer survival (52). Polymorphisms in CYP3A5 (a cytochrome P450 family member) can define a subset of BCa patients who better respond to cabazitaxel and temsirolimus, in terms of lower toxicity and higher efficacy (53, 54).

However, regarding the last two studies (19, 20), it should be noted that WGCNA was performed using sample sizes that were too small, that is 8, 9, or 11 samples for each condition. Indeed, at least 20 samples are required for a robust WGCNA analysis and to obtain reliable results.

Recently, also miRNA expression data of 418 BCa and 19 normal tissue samples collected in The Cancer Genome Atlas (TCGA) have been investigated (21). After the selection of differentially expressed miRNAs, WGCNA analysis allowed the identification of a module closely related to BCa progression. Thirteen downregulated miRNAs (Table 1) also had a prognostic value since their low expression levels were associated with poorer OS in BCa patients. Interestingly, the predicted targets of these miRNAs were found to be significantly enriched in cancer-related pathways (Table 1). It has been shown that miR-1-1 and miR-133a are under-expressed in BCa cells and act as tumor suppressive miRNAs since, when overexpressed, they inhibited cell proliferation and invasion and increased apoptosis (55). In particular, miR-1 can exert its tumor suppressive function by targeting both coding genes, for example CCL2 (56), and non-coding RNAs, including UCA1 (57). MiR-133a can also induce apoptosis through silencing of GSTP1 in BCa cell lines (58) and, along with miR-1, it can inhibit BCa cell proliferation and increase apoptosis by targeting TAGLN2 mRNA (59). Moreover, miR-133a and miR-145 suppress cancer cell proliferation by directly regulating FSCN1 expression (60). MiR-133b plays a key role in proliferation and apoptosis by silencing Bcl-W and AKT1 genes (61) and its downregulation is associated with BCa progression and poor prognosis (62). Also miR-139 is a tumor suppressive miRNA since it can inhibit BCa cell proliferation by targeting the BMI1 oncogene (63) and cell migration and invasion by silencing matrix metalloprotease 11 (MMP11) (64). It has been observed that miR-143 can inhibit tumor cell proliferation, it is associated with BCa resistance to gemcitabine (65) and, along with miR-145, is a good prognostic biomarker for BCa patient survival (66). Interestingly, the polymorphism rs353293 in the common promoter of miR-143 and miR-145 is associated with BCa risk (67). Finally, miR-195 suppressed cancer cell proliferation by silencing GLUT3 (68), CDK4 (69), and CDC42 (70) genes. Therefore, these tumor suppressive miRNAs can be considered for *in vivo* delivery of therapeutics in bladder cancer, even though there are still challenges to the development of miRNA delivery strategies without toxicity induction.

## Further analyses on identified hub genes and miRNAs

We performed a Gene Ontology analysis using all hub genes identified by WGCNA in the reviewed studies. Overall, they were found to belong to related biological processes. In particular, we highlighted pathways such as cell cycle, mitosis, mitotic spindle organization, kinetochore assembly, and nuclear division. All these processes are involved in the uncontrolled cell proliferation in cancers. In addition, by using the transcription factor binding data generated by the ENCODE project consortium (www.encodeproject.org), we identified the E2F4 and FOXM1 transcription factors as the master regulators of hub gene expression, since they resulted as being significantly over-represented in the promoters of hub genes (*p* = 5.892e-9 and *p* = 3.220e-8, respectively). Moreover, in order to investigate whether relationships exist between these miRNAs and the hub genes listed in Table 1, we performed a comprehensive literature search and identified which hub genes are targets of the hub miRNAs. In order to increase the analysis stringency, we considered only the experimentally assessed miRNA targets. Results reported in Table 2 show that nearly all hub miRNAs target at least one hub gene, therefore these miRNAs could be involved in the deregulation of hub genes. Alternatively, hub miRNAs and genes could be deregulated due to the alteration of master regulators, such as transcription factors.

**Table 2:** Experimentally validated targets (among BCa hub genes previously identified by WGCNA) of hub miRNAs recently identified in BCa (21).

**Hub miRNAs**	**Targeted hub genes**	**Validation assay**	**Cell lines**	**References**
miR-1-1/2	CENPF	Proteomics (pSILAC)	HeLa (human cervical cancer)	(71)
	FBN1			
	HERC2			
	KIF2C			
	KIF4A			
	UBE2I			
miR-28	PTPRJ	PAR-CLIP	HCT116 (human colon cancer)	(72)
miR-133a-1/2	CDCA8	PAR-CLIP	C8166 (HIV-1 infected human T cells) and TZM-bl (HIV-1 infected human epithelial cells)	(73)
	FBN1	Microarray	T24 and KK47 (human bladder cancer)	(58)
miR-139	MMP11	Luciferase reporter assay, Microarray, qRT-PCR, Western blot	T24 and BOY (human bladder cancer)	(64)
miR-143	BRD2	PAR-CLIP	HCT116 (human colon cancer)	(72)
	CDC25B			
	COL3A1	*In situ* hybridization, qRT-PCR, Western blot	NF-38 (human normal gastric fibroblasts) and CaF-38 (human cancer-associated fibroblasts)	(74)
	COL5A2	Microarray	USSCs (human unrestricted somatic stem cells)	(75)
miR-195	CEP55	PAR-CLIP	HEK293S (human embryonic kidney) and MCF7 (breast cancer)	(76–79)
	PTPRJ	HITS-CLIP	Jijoye (EBV-transformed B cells)	(80)
	PURA			
	TRAK1	HITS-CLIP	HEK293S (human embryonic kidney) and fresh-frozen human heart tissues	(81, 82)
	UBE2I	Microarray, qRT-PCR	PBMCs (peripheral blood mononuclear blood cells)	(83)
miR-6507	CDC25B	PAR-CLIP	HCT116 (human colon cancer)	(72)
	CDCA8			
	DCAF8	PAR-CLIP	HEK293S (human embryonic kidney)	(76)
	PRC1			
	CENPA	PAR-CLIP	hESCs (human embryonic stem cells)	(84)
	CEP55	PAR-CLIP	BC-1 and BC-3 (KSHV-infected primary effusion lymphoma)	(85)

*pSILAC, pulsed Stable Isotope Labeling with Aminoacids in Cell culture; PAR-CLIP, PhotoActivatable Ribonucleoside-enhanced CrossLinking and ImmunoPrecipitation; HITS-CLIP, HIgh-Throughput Sequencing of RNA isolated by CrossLinking and ImmunoPrecipitation*.

## Conclusions

WGCNA is a recently developed method for the analysis of gene expression data able to propose candidate therapeutic targets or diagnostic/prognostic biomarkers. Here, we reviewed all studies where WGCNA has been applied for the analysis of expression data from BCa. They include analyses of gene and miRNA expression data. Notably, neither lncRNA expression or splicing isoform-specific RNA expression have yet been investigated in BCa by WGCNA, as recently carried out, for example, in pancreatic cancer (15) and in clear cell renal cell carcinoma (ccRCC) (86), respectively. In addition, expression data of circular RNAs and RNAs in exosomes have not been analyzed by WGCNA in any cancer type. Recently, this network strategy has also been applied to proteomic and metabolomic data, although, due to the low coverage of proteomic and metabolomic analytical methods, WGCNA needed to be modified (87). Moreover, since RNA-seq data simultaneously allow the gene expression measure and mutation analysis, it would be interesting to perform an evaluation of the mutation effects on the gene co-expression module assignment of a gene.

Although WGCNA requires expression data from at least 20 samples in order to obtain reliable results, only 8, 9, or 11 samples for each condition were analyzed in two reviewed studies (19, 20). Generally, in order to overcome the problem of a small sample size, researchers use more than one expression dataset, particularly during analysis of microarray data. However, systematic and technical differences between different microarray platforms and datasets (called batch effects) could emerge. The correct data pre-processing is needed since WGCNA is sensitive to batch effects. Moreover, the presence of outliers (samples with very different expression profiles form the bulk) may affect WGCNA results, thus their removal is a critical step (12). Unfortunately, sometimes researchers tend to reject few outliers because of the small sample size. A further critical element for WGNCA analysis is the highly variable gene expression among samples due to tumor heterogeneity. To overcome this problem, large sample size expression datasets should be used and, in particular, datasets that include samples isolated from different points of a single cancer tissue should be preferred. Moreover, we suggest performing sample clustering based on expression data before WGCNA analysis, in order to process less heterogeneous samples, as recently carried out for ccRCC (88).

High BCa heterogeneity, different microarray platforms, specific setting of WGCNA algorithm and different patient therapies, lifestyle, stages, age, and sex could explain the little overlap between key genes identified among the studies. However, since these hub genes are highly co-expressed, they could highlight a common mechanism of transcriptional and post-transcriptional regulation, thus revealing mechanistic insights into cancer development. In particular, we identified the hub gene FOXM1 as the master regulator of the other genes identified by WGCNA. Hub genes could also be related at a functional level, since they belong to similar pathways and are regulated by a few common hub miRNAs. Furthermore, these miRNAs are known to be involved in the same pathways of hub genes, thus supporting the role in BCa of genes and miRNAs identified by WGCNA. Finally, although currently no hub gene has sufficient validity for clinical practice, many of them are already known to serve as biomarkers in BCa. Therefore, these genes are worth being further explored, since they can shed light into the molecular mechanisms of BCa, thus leading to the definition of novel personalized therapies. For example, regarding biomarkers for chemotherapy efficacy and toxicity, WGCNA identified the cytochrome P450 family member CYP3A5, already found to be useful in defining BCa patients with better responses to treatments.

## Author contributions

MG and FP conception and design. MG, GO, ARi, and FP drafting the manuscript. GO, ARi, MB, and AC review of the literature. ARu, EC, TC, and GP critical revision of the manuscript.

### Conflict of interest statement

The authors declare that the research was conducted in the absence of any commercial or financial relationships that could be construed as a potential conflict of interest. The handling Editor declared a shared affiliation, though no other collaboration, with several of the authors MG, GO, ARi, MB, TC, GP and FP.

## References

[1] Antoni S Ferlay J Soerjomataram I Znaor A Jemal A Bray F. Bladder cancer incidence and mortality: a global overview and recent trends. Eur Urol. (2017) 71:96–108. 10.1016/j.eururo.2016.06.010 27370177

[2] Galsky MD. Bladder cancer in 2017: Advancing care through genomics and immune checkpoint blockade. Nat Rev Urol. (2018) 15:71–2. 10.1038/nrurol.2017.199 29182606

[3] Sanli O Dobruch J Knowles MA Burger M Alemozaffar M Nielsen ME. Bladder cancer. Nat Rev Dis Primers (2017) 3:17022. 10.1038/nrdp.2017.22 28406148

[4] Lotan Y Elias K Svatek RS Bagrodia A Nuss G Moran B. Bladder cancer screening in a high risk asymptomatic population using a point of care urine based protein tumor marker. J Urol. (2009) 182:52–7; discussion 58. 10.1016/j.juro.2009.02.142 19450825

[5] Leiblich A. Recent developments in the search for urinary biomarkers in bladder cancer. Curr Urol Rep. (2017) 18:100. 10.1007/s11934-017-0748-x 29130146 PMC5682315

[6] Giulietti M Santoni M Cimadamore A Carrozza F Piva F Cheng L. Exploring small extracellular vesicles for precision medicine in prostate cancer. Front Oncol. (2018) 8:221. 10.3389/fonc.2018.00221 29951374 PMC6008382

[7] Nawaz M Camussi G Valadi H Nazarenko I Ekstrom K Wang X. The emerging role of extracellular vesicles as biomarkers for urogenital cancers. Nat Rev Urol. (2014) 11:688–701. 10.1038/nrurol.2014.301 25403245

[8] Barabasi AL Oltvai ZN. Network biology: understanding the cell's functional organization. Nat Rev Genet. (2004) 5:101–13. 10.1038/nrg1272 14735121

[9] de la Fuente A From “differential expression” to “differential networking” - identification of dysfunctional regulatory networks in diseases. Trends Genet. (2010) 26:326–33. 10.1016/j.tig.2010.05.001 20570387

[10] Zeng T Sun SY Wang Y Zhu H Chen L. Network biomarkers reveal dysfunctional gene regulations during disease progression. FEBS J. (2013) 280:5682–95. 10.1111/febs.12536 24107168

[11] Langfelder P Horvath S. WGCNA: an R package for weighted correlation network analysis. BMC Bioinformatics (2008) 9:559. 10.1186/1471-2105-9-559 19114008 PMC2631488

[12] Oldham MC Konopka G Iwamoto K Langfelder P Kato T Horvath S. Functional organization of the transcriptome in human brain. Nat Neurosci. (2008) 11:1271–82. 10.1038/nn.2207 18849986 PMC2756411

[13] Giulietti M Occhipinti G Principato G Piva F. Identification of candidate miRNA biomarkers for pancreatic ductal adenocarcinoma by weighted gene co-expression network analysis. Cell Oncol. (2017) 40:181–92. 10.1007/s13402-017-0315-y 28205147 PMC13001553

[14] Giulietti M Occhipinti G Principato G Piva F. Weighted gene co-expression network analysis reveals key genes involved in pancreatic ductal adenocarcinoma development. Cell Oncol. (2016) 39:379–88. 10.1007/s13402-016-0283-7 27240826 PMC13001876

[15] Giulietti M Righetti A Principato G Piva F. LncRNA co-expression network analysis reveals novel biomarkers for pancreatic cancer. Carcinogenesis (2018) 39:1016–25. 10.1093/carcin/bgy069 29796634

[16] Yuan L Shu B Chen L Qian K Wang Y Qian G. Overexpression of COL3A1 confers a poor prognosis in human bladder cancer identified by co-expression analysis. Oncotarget (2017) 8:70508–20. 10.18632/oncotarget.19733 29050298 PMC5642573

[17] Li S Liu X Liu T Meng X Yin X Fang C. Identification of biomarkers correlated with the TNM staging and overall survival of patients with bladder cancer. Front Physiol. (2017) 8:947. 10.3389/fphys.2017.00947 29234286 PMC5712410

[18] Zhang X Zhang M Hou Y Xu L Li W Zou Z. Single-cell analyses of transcriptional heterogeneity in squamous cell carcinoma of urinary bladder. Oncotarget (2016) 7:66069–76. 10.18632/oncotarget.11803 27602771 PMC5323215

[19] Deng SP Zhu L Huang DS. Mining the bladder cancer-associated genes by an integrated strategy for the construction and analysis of differential co-expression networks. BMC Genomics (2015) 16(Suppl. 3):S4. 10.1186/1471-2164-16-S3-S4 25707808 PMC4331807

[20] Gaballah HH Integration of gene coexpression network, GO enrichment analysis for identification gene expression signature of invasive bladder carcinoma. Transcriptomics (2016) 4:126 10.4172/2329-8936.1000126

[21] Zhao F Ge YZ Zhou LH Xu LW Xu Z Ping WW. Identification of hub miRNA biomarkers for bladder cancer by weighted gene coexpression network analysis. Onco Targets Ther. (2017) 10:5551–9. 10.2147/OTT.S146479 29200870 PMC5702163

[22] Kim CJ Yoshioka N Tambe Y Kushima R Okada Y Inoue H. Periostin is down-regulated in high grade human bladder cancers and suppresses *in vitro* cell invasiveness and *in vivo* metastasis of cancer cells. Int J Cancer (2005) 117:51–8. 10.1002/ijc.21120 15880581

[23] Kim CJ Sakamoto K Tambe Y Inoue H. Opposite regulation of epithelial-to-mesenchymal transition and cell invasiveness by periostin between prostate and bladder cancer cells. Int J Oncol. (2011) 38:1759–66. 10.3892/ijo.2011.997 21468544

[24] Bufo P Sanguedolce F Tortorella S Cormio L Carrieri G Pannone G. Expression of mitotic kinases phospho-aurora A and aurora B correlates with clinical and pathological parameters in bladder neoplasms. Histol Histopathol. (2010) 25:1371–7. 10.14670/HH-25.1371 20865660

[25] Yamamoto Y Matsuyama H Chochi Y Okuda M Kawauchi S Inoue R. Overexpression of BUBR1 is associated with chromosomal instability in bladder cancer. Cancer Genet Cytogenet. (2007) 174:42–7. 10.1016/j.cancergencyto.2006.11.012 17350465

[26] Zhang Z Zhang G Kong C. High expression of Cdc25B and low expression of 14-3-3sigma is associated with the development and poor prognosis in urothelial carcinoma of bladder. Tumour Biol. (2014) 35:2503–12. 10.1007/s13277-013-1331-9 24234332

[27] Lei CY Wang W Zhu YT Fang WY Tan WL. The decrease of cyclin B2 expression inhibits invasion and metastasis of bladder cancer. Urol Oncol. (2016) 34:237 e1–10. 10.1016/j.urolonc.2015.11.011 26706119

[28] Rinaldetti S Wirtz RM Worst TS Eckstein M Weiss CA Breyer J. FOXM1 predicts overall and disease specific survival in muscle-invasive urothelial carcinoma and presents a differential expression between bladder cancer subtypes. Oncotarget (2017) 8:47595–606. 10.18632/oncotarget.17394 28498805 PMC5564590

[29] Liu D Zhang Z Kong CZ. High FOXM1 expression was associated with bladder carcinogenesis. Tumour Biol. (2013) 34:1131–8. 10.1007/s13277-013-0654-x 23325617

[30] Inoguchi S Seki N Chiyomaru T Ishihara T Matsushita R Mataki H. Tumour-suppressive microRNA-24-1 inhibits cancer cell proliferation through targeting FOXM1 in bladder cancer. FEBS Lett. (2014) 588:3170–9. 10.1016/j.febslet.2014.06.058 24999187

[31] Li WM Wei YC Huang CN Ke HL Li CC Yeh HC. Matrix metalloproteinase-11 as a marker of metastasis and predictor of poor survival in urothelial carcinomas. J Surg Oncol. (2016) 113:700–7. 10.1002/jso.24195 26861489

[32] Zhang J Jia Q Zou S Zhang P Zhang X Skog S. Thymidine kinase 1: a proliferation marker for determining prognosis and monitoring the surgical outcome of primary bladder carcinoma patients. Oncol Rep. (2006) 15:455–61. 10.3892/or.15.2.455 16391869

[33] Kim YH Yan C Lee IS Piao XM Byun YJ Jeong P. Value of urinary topoisomerase-IIA cell-free DNA for diagnosis of bladder cancer. Investig Clin Urol. (2016) 57:106–12. 10.4111/icu.2016.57.2.106 26981592 PMC4791669

[34] Kim EJ Lee YS Kim YJ Kim MJ Ha YS Jeong P. Clinical implications and prognostic values of topoisomerase-II alpha expression in primary non-muscle-invasive bladder cancer. Urology (2010) 75:1516 e9–13. 10.1016/j.urology.2009.08.055 19913893

[35] Raspollini MR Luque RJ Menendez CL Bollito E Brunelli M Martignoni G. T1 high-grade bladder carcinoma outcome: the role of p16, topoisomerase-IIalpha, survivin, and E-cadherin. Hum Pathol. (2016) 57:78–84. 10.1016/j.humpath.2016.06.022 27473264

[36] Yan L Li S Xu C Zhao X Hao B Li H. Target protein for Xklp2 (TPX2), a microtubule-related protein, contributes to malignant phenotype in bladder carcinoma. Tumour Biol. (2013) 34:4089–100. 10.1007/s13277-013-1000-z 23873098

[37] Morikawa T Kawai T Abe H Kume H Homma Y Fukayama M. UBE2C is a marker of unfavorable prognosis in bladder cancer after radical cystectomy. Int J Clin Exp Pathol. (2013) 6:1367–74. 23826418 PMC3693202

[38] Fristrup N Birkenkamp-Demtroder K Reinert T Sanchez-Carbayo M Segersten U Malmstrom PU. Multicenter validation of cyclin D1, MCM7, TRIM29, and UBE2C as prognostic protein markers in non-muscle-invasive bladder cancer. Am J Pathol. (2013) 182:339–49. 10.1016/j.ajpath.2012.10.017 23201130

[39] Zheng M Simon R Mirlacher M Maurer R Gasser T Forster T. TRIO amplification and abundant mRNA expression is associated with invasive tumor growth and rapid tumor cell proliferation in urinary bladder cancer. Am J Pathol. (2004) 165:63–9. 10.1016/S0002-9440(10)63275-0 15215162 PMC1618551

[40] Yamamoto Y Suehiro Y Suzuki A Nawata R Kawai Y Inoue R. Germline DNA copy number variations as potential prognostic markers for non-muscle invasive bladder cancer progression. Oncol Lett. (2017) 14:1193–9. 10.3892/ol.2017.6233 28693295 PMC5494660

[41] Zhang Z Huettner PC Nguyen L Bidder M Funk MC Li J. Aberrant promoter methylation and silencing of the POU2F3 gene in cervical cancer. Oncogene (2006) 25:5436–45. 10.1038/sj.onc.1209530 16607278

[42] Sun WX Chen YH Liu ZZ Xie JJ Wang W Du YP. Association between the CYP1A2 polymorphisms and risk of cancer: a meta-analysis. Mol Genet Genomics (2015) 290:709–25. 10.1007/s00438-014-0956-8 25472037

[43] Piva F Giulietti M Burini AB Principato G. SpliceAid 2: a database of human splicing factors expression data and RNA target motifs. Hum Mutat. (2012) 33:81–5. 10.1002/humu.21609 21922594

[44] Piva F Giulietti M Nocchi L Principato G. SpliceAid: a database of experimental RNA target motifs bound by splicing proteins in humans. Bioinformatics (2009) 25:1211–3. 10.1093/bioinformatics/btp124 19261717

[45] Giulietti M Piva F D'Antonio M D'Onorio De Meo P Paoletti D Castrignano T. SpliceAid-F: a database of human splicing factors and their RNA-binding sites. Nucleic Acids Res. (2013) 41:D125–31. 10.1093/nar/gks997 23118479 PMC3531144

[46] Piva F Giulietti M Nardi B Bellantuono C Principato G. An improved *in silico* selection of phenotype affecting polymorphisms in SLC6A4, HTR1A and HTR2A genes. Hum Psychopharmacol. (2010) 25:153–61. 10.1002/hup.1100 20196180

[47] Piva F Giulietti M Baldelli L Nardi B Bellantuono C Armeni T. Bioinformatic analyses to select phenotype affecting polymorphisms in HTR2C gene. Hum Psychopharmacol. (2011) 26:365–72. 10.1002/hup.1214 21717509

[48] Piva F Giulietti M Occhipinti G Santoni M Massari F Sotte V. Computational analysis of the mutations in BAP1, PBRM1 and SETD2 genes reveals the impaired molecular processes in renal cell carcinoma. Oncotarget (2015) 6:32161–8. 10.18632/oncotarget.5147 26452128 PMC4741666

[49] Giulietti M Milantoni SA Armeni T Principato G Piva F. ExportAid: database of RNA elements regulating nuclear RNA export in mammals. Bioinformatics (2015) 31:246–51. 10.1093/bioinformatics/btu620 25273107

[50] Piva F Giulietti M Armeni T Principato G. Cross-link immunoprecipitation data to detect polymorphisms lying in splicing regulatory motifs: a method to refine single nucleotide polymorphism selection in association studies. Psychiatr Genet. (2012) 22:88–91. 10.1097/YPG.0b013e32834c0bd1 21946076

[51] Giulietti M Grillo G Liuni S Pesole G. A guideline for the annotation of UTR regulatory elements in the UTRsite collection. Methods Mol Biol. (2015) 1269:339–48. 10.1007/978-1-4939-2291-8_21 25577389

[52] Xie JY Chen PC Zhang JL Gao ZS Neves H Zhang SD. The prognostic significance of DAPK1 in bladder cancer. PLoS ONE (2017) 12:e0175290. 10.1371/journal.pone.0175290 28388658 PMC5384764

[53] Duran I Hagen C Arranz JA Apellaniz-Ruiz M Perez-Valderrama B Sala N. SNPs associated with activity and toxicity of cabazitaxel in patients with advanced urothelial cell carcinoma. Pharmacogenomics (2016) 17:463–71. 10.2217/pgs.15.186 27020167

[54] Mbatchi LC Gassiot M Pourquier P Goberna A Mahammedi H Mourey L. Association of NR1I2, CYP3A5 and ABCB1 genetic polymorphisms with variability of temsirolimus pharmacokinetics and toxicity in patients with metastatic bladder cancer. Cancer Chemother Pharmacol. (2017) 80:653–9. 10.1007/s00280-017-3379-5 28676933

[55] Yamasaki T Yoshino H Enokida H Hidaka H Chiyomaru T Nohata N. Novel molecular targets regulated by tumor suppressors microRNA-1 and microRNA-133a in bladder cancer. Int J Oncol. (2012) 40:1821–30. 10.3892/ijo.2012.1391 22378464

[56] Wang W Shen F Wang C Lu W Wei J Shang A. MiR-1-3p inhibits the proliferation and invasion of bladder cancer cells by suppressing CCL2 expression. Tumour Biol. (2017) 39:1010428317698383. 10.1177/1010428317698383 28618950

[57] Wang T Yuan J Feng N Li Y Lin Z Jiang Z. Hsa-miR-1 downregulates long non-coding RNA urothelial cancer associated 1 in bladder cancer. Tumour Biol. (2014) 35:10075–84. 10.1007/s13277-014-2321-2 25015192

[58] Uchida Y Chiyomaru T Enokida H Kawakami K Tatarano S Kawahara K. MiR-133a induces apoptosis through direct regulation of GSTP1 in bladder cancer cell lines. Urol Oncol. (2013) 31:115–23. 10.1016/j.urolonc.2010.09.017 21396852

[59] Yoshino H Chiyomaru T Enokida H Kawakami K Tatarano S Nishiyama K. The tumour-suppressive function of miR-1 and miR-133a targeting TAGLN2 in bladder cancer. Br J Cancer (2011) 104:808–18. 10.1038/bjc.2011.23 21304530 PMC3048214

[60] Chiyomaru T Enokida H Tatarano S Kawahara K Uchida Y Nishiyama K. miR-145 and miR-133a function as tumour suppressors and directly regulate FSCN1 expression in bladder cancer. Br J Cancer (2010) 102:883–91. 10.1038/sj.bjc.6605570 20160723 PMC2833258

[61] Chen XN Wang KF Xu ZQ Li SJ Liu Q Fu DH. MiR-133b regulates bladder cancer cell proliferation and apoptosis by targeting Bcl-w and Akt1. Cancer Cell Int. (2014) 14:70. 10.1186/s12935-014-0070-3 25414595 PMC4238049

[62] Chen X Wu B Xu Z Li S Tan S Liu X. Downregulation of miR-133b predict progression and poor prognosis in patients with urothelial carcinoma of bladder. Cancer Med. (2016) 5:1856–62. 10.1002/cam4.777 27292588 PMC4971914

[63] Luo H Yang R Li C Tong Y Fan L Liu X. MicroRNA-139-5p inhibits bladder cancer proliferation and self-renewal by targeting the Bmi1 oncogene. Tumour Biol. (2017) 39:1010428317718414. 10.1177/1010428317718414 28720065

[64] Yonemori M Seki N Yoshino H Matsushita R Miyamoto K Nakagawa M. Dual tumor-suppressors miR-139-5p and miR-139-3p targeting matrix metalloprotease 11 in bladder cancer. Cancer Sci. (2016) 107:1233–42. 10.1111/cas.13002 27355528 PMC5021030

[65] Wang H Li Q Niu X Wang G Zheng S Fu G. miR-143 inhibits bladder cancer cell proliferation and enhances their sensitivity to gemcitabine by repressing IGF-1R signaling. Oncol Lett. (2017) 13:435–40. 10.3892/ol.2016.5388 28123579 PMC5245146

[66] Avgeris M Mavridis K Tokas T Stravodimos K Fragoulis EG Scorilas A. Uncovering the clinical utility of miR-143, miR-145 and miR-224 for predicting the survival of bladder cancer patients following treatment. Carcinogenesis (2015) 36:528–37. 10.1093/carcin/bgv024 25804644

[67] Wu J Huang Q Meng D Huang M Li C Qin T. A functional rs353293 polymorphism in the promoter of miR-143/145 is associated with a reduced risk of bladder cancer. PLoS ONE (2016) 11:e0159115. 10.1371/journal.pone.0159115 27438131 PMC4954649

[68] Fei X Qi M Wu B Song Y Wang Y Li T. MicroRNA-195-5p suppresses glucose uptake and proliferation of human bladder cancer T24 cells by regulating GLUT3 expression. FEBS Lett. (2012) 586:392–7. 10.1016/j.febslet.2012.01.006 22265971

[69] Lin Y Wu J Chen H Mao Y Liu Y Mao Q. Cyclin-dependent kinase 4 is a novel target in micoRNA-195-mediated cell cycle arrest in bladder cancer cells. FEBS Lett. (2012) 586:442–7. 10.1016/j.febslet.2012.01.027 22289176

[70] Zhao C Qi L Chen M Liu L Yan W Tong S. microRNA-195 inhibits cell proliferation in bladder cancer via inhibition of cell division control protein 42 homolog/signal transducer and activator of transcription-3 signaling. Exp Ther Med. (2015) 10:1103–8. 10.3892/etm.2015.2633 26622447 PMC4533204

[71] Selbach M Schwanhausser B Thierfelder N Fang Z Khanin R Rajewsky N. Widespread changes in protein synthesis induced by microRNAs. Nature (2008) 455:58–63. 10.1038/nature07228 18668040

[72] Krell J Stebbing J Carissimi C Dabrowska AF de Giorgio A Frampton AE. TP53 regulates miRNA association with AGO2 to remodel the miRNA-mRNA interaction network. Genome Res. (2016) 26:331–41. 10.1101/gr.191759.115 26701625 PMC4772015

[73] Whisnant AW Bogerd HP Flores O Ho P Powers JG Sharova N. In-depth analysis of the interaction of HIV-1 with cellular microRNA biogenesis and effector mechanisms. MBio (2013) 4:e000193. 10.1128/mBio.00193-13 23592263 PMC3634607

[74] Naito Y Sakamoto N Oue N Yashiro M Sentani K Yanagihara K. MicroRNA-143 regulates collagen type III expression in stromal fibroblasts of scirrhous type gastric cancer. Cancer Sci. (2014) 105:228–35. 10.1111/cas.12329 24283360 PMC4317817

[75] Bakhshandeh B Soleimani M Paylakhi SH Ghaemi N. A microRNA signature associated with chondrogenic lineage commitment. J Genet. (2012) 91:171–82. 22942087 10.1007/s12041-012-0168-0

[76] Hafner M Landthaler M Burger L Khorshid M Hausser J Berninger P. Transcriptome-wide identification of RNA-binding protein and microRNA target sites by PAR-CLIP. Cell (2010) 141:129–41. 10.1016/j.cell.2010.03.009 20371350 PMC2861495

[77] Farazi TA Ten Hoeve JJ Brown M Mihailovic A Horlings HM van de Vijver MJ. Identification of distinct miRNA target regulation between breast cancer molecular subtypes using AGO2-PAR-CLIP and patient datasets. Genome Biol. (2014) 15:R9. 10.1186/gb-2014-15-1-r9 24398324 PMC4053773

[78] Memczak S Jens M Elefsinioti A Torti F Krueger J Rybak A. Circular RNAs are a large class of animal RNAs with regulatory potency. Nature (2013) 495:333–8. 10.1038/nature11928 23446348

[79] Kishore S Jaskiewicz L Burger L Hausser J Khorshid M Zavolan M. A quantitative analysis of CLIP methods for identifying binding sites of RNA-binding proteins. Nat Methods (2011) 8:559–64. 10.1038/nmeth.1608 21572407

[80] Riley KJ Rabinowitz GS Yario TA Luna JM Darnell RB Steitz JA. EBV and human microRNAs co-target oncogenic and apoptotic viral and human genes during latency. EMBO J. (2012) 31:2207–21. 10.1038/emboj.2012.63 22473208 PMC3343464

[81] Karginov FV Hannon GJ. Remodeling of Ago2-mRNA interactions upon cellular stress reflects miRNA complementarity and correlates with altered translation rates. Genes Dev. (2013) 27:1624–32. 10.1101/gad.215939.113 23824327 PMC3731550

[82] Spengler RM Zhang X Cheng C McLendon JM Skeie JM Johnson FL. Elucidation of transcriptome-wide microRNA binding sites in human cardiac tissues by Ago2 HITS-CLIP. Nucleic Acids Res. (2016) 44:7120–31. 10.1093/nar/gkw640 27418678 PMC5009757

[83] Devadas K Biswas S Haleyurgirisetty M Ragupathy V Wang X Lee S. Identification of host micro RNAs that differentiate HIV-1 and HIV-2 infection using genome expression profiling techniques. Viruses (2016) 8:E12. 10.3390/v8050121 27144577 PMC4885076

[84] Lipchina I Elkabetz Y Hafner M Sheridan R Mihailovic A Tuschl T. Genome-wide identification of microRNA targets in human ES cells reveals a role for miR-302 in modulating BMP response. Genes Dev. (2011) 25:2173–86. 10.1101/gad.17221311 22012620 PMC3205587

[85] Gottwein E Corcoran DL Mukherjee N Skalsky RL Hafner M Nusbaum JD. Viral microRNA targetome of KSHV-infected primary effusion lymphoma cell lines. Cell Host Microbe (2011) 10:515–26. 10.1016/j.chom.2011.09.012 22100165 PMC3222872

[86] Hamilton MJ Girke T Martinez E. Global isoform-specific transcript alterations and deregulated networks in clear cell renal cell carcinoma. Oncotarget (2018) 9:23670–80. 10.18632/oncotarget.25330 29805765 PMC5955119

[87] Pei G Chen L Zhang W. WGCNA application to proteomic and metabolomic data analysis. Methods Enzymol. (2017) 585:135–58. 10.1016/bs.mie.2016.09.016 28109426

[88] Wu P Liu JL Pei SM Wu CP Yang K Wang SP. Integrated genomic analysis identifies clinically relevant subtypes of renal clear cell carcinoma. BMC Cancer (2018) 18:287. 10.1186/s12885-018-4176-1 29534679 PMC5851245

